# Estimating the Social Costs of Child Abuse in Residential Care for Children with Disabilities Using the Japanese Survey on the Interactions of Adverse and Positive Childhood Experiences toward Adulthood

**DOI:** 10.3390/ijerph192416476

**Published:** 2022-12-08

**Authors:** Naruhisa Nakane, Ichiro Wada

**Affiliations:** 1 Department of Public Policy, Faculty of Welfare Society, Kyoto Prefectural University, 1-5, Shimogamohangi-cyo, Sakyo-ku, Kyoto 606-8522, Japan; 2 Department of Interdisciplinary Studies, Faculty of International Liberal Arts, Dokkyo University, 1-1, Gakuen-cho, Soka-shi 340-0042, Japan

**Keywords:** child maltreatment, child abuse, social costs of child abuse, residential care for children with disabilities, Japan

## Abstract

We sought to calculate the extra social costs resulting from child abuse in residential care facilities (RCFs) for children with disabilities (CWD) in Japan. We distributed a survey to 260 residential facilities for CWD in 2020 and obtained responses from 91 facilities. Among the children placed in these facilities, our estimates by four different criteria determined that 23–67% were affected by child abuse. We also estimated extra costs for each of the four criteria, which we estimated to average USD 647.7 million. This study is meaningful in that there are no existing official statistics or research findings on the extra costs of residential care due to child abuse in Japan.

## 1. Introduction

### 1.1. Current Situation of Residential Institutions for Japanese Children with Disabilities

This study aimed to calculate the additional social costs stemming from child abuse in the expenses of Japanese residential care facilities (RCFs) for children with disabilities (CWD). This project was initially undertaken by Wada and Igarashi to determine the additional costs of Japanese child maltreatment, but these researchers did not calculate the costs of residential care for CWD [1]. Complimenting their research, our study provides a further precise view of the extra social costs of Japanese child abuse. Hughes et al. examined the relationship between adverse childhood experiences (ACEs), health, and other types of financial expenses in 28 European nations, similar to our study [2]. Meanwhile, Isumi et al. constructed medical assumptions about the older Japanese population with inappropriate childhood treatment and suggested that such childhood experiences can impact an individual’s life and generate social expenses over the long term [3].

An investigation into this subject reveals enormous annual additional costs incurred from child abuse, incentivizing governments and local authorities to reduce child abuse in society.

Established under Article 25 of the Child Welfare Act [4], Japanese institutions for CWD are operated to provide protection, teach regular life skills, and provide knowledge to CWD. Two types of institutions exist: medical institutions for children with physical or severe multiple disabilities, and welfare institutions for children with intellectual disabilities, behavioral disorders, or developmental disabilities. The number of children admitted to these institutions has remained unchanged or is in decline. Although RCFs for CWD were originally intended for children with congenital impairments, such as intellectual disabilities, autistic spectrum disorder, and severe physical conditions, government research found that the primary reason for admission is behavioral disorders caused by post-traumatic stress disorder (PTSD), secondary to inappropriate treatment from caregivers (i.e., maltreatment).

The Ministry of Health, Labour and Welfare’s (MHLW) Department of Health and Welfare for Persons with Disabilities (DHWPD) classifies the functions of RCFs for CWD as developmental, independence, and communal support, in addition to social care [5]. As for social care, Tsuru pointed out that the four kinds of infant homes—foster homes, maternal and child living support facilities, and foster parents—comprise 41% of the route of admission to RCFs for CWD, and a substantial portion of the reasons for admission are due to social care [6] (p. 21). Social care refers to care provided by the central government, local governments, or other entities in the public sector to children who require care in their family environment, such as when a child has no guardian or has difficulty obtaining care from a guardian due to abuse or other reasons. As of 2022, approximately 42,000 people receive social care in Japan, including 6019 children adopted by foster parents, 1688 children in small facilities, and 34,727 children in regular facilities. Approximately 80% of children who leave social care live in institutions. This is a major policy issue, as the full guarantee of children’s rights has not yet been realized. Suzuki indicated that the share of abused children is rising in RCFs for CWD, implying that an extra cost of abuse is occurring in these institutions [7].

### 1.2. Overage Children (Ka-Rei-Ji)

RCFs for CWD in Japan accept children with the expectation of providing social care. Although RCFs are essentially expected to function as temporary substitutes for home care, parents are unlikely to recover their childcare functions, and the placement is likely to be prolonged. It is unlikely that physically disabled children or those with severe intellectual disabilities or autistic tendencies—who need assistance in all aspects of life—will be able to live at home or in the community, even when they grow up and become legal adults at age 18. As a result, such children will continue on to residential facilities for adults, which are usually located at the same sites as RCFs for CWD, despite the fact that other young people of the same generation typically attend day centers away from their parents. In Japan, these adults are called ka-rei-ji (overage children). MHLW, which has jurisdiction over them, is aware of this issue. As such, the Study Group on the State of Residential Institutions for Children with Disabilities, which is run by the DHWPD, was established; its March 2020 report [5] underscores the strengthening of independence support functions for overage children as a task for RCFs for CWD. This has long been a problem but has yet to be resolved. One reason is that once a child is placed in an RCF, family and community ties are severed, making it difficult for the child to leave the facility and forcing them to make the facility the foundation of their life on a continual basis. As of 2020, there are 1500 overage children in welfare institutions for CWD and 18,141 in medical institutions for CWD, amounting to 19,641. The large number of overage children in medical institutions indicates the lack of a stable medical/care system. In Japan, the Diet passed the Act on Support for Children with Medical Care and their Families in 2021. However, this implies that the development of a stable, ongoing medical/care system in the community has only just begun.

### 1.3. Prior Studies on Extra Costs

The concept of extra costs refers to the monetary costs or losses incurred compared to if the causative factor or phenomenon had not occurred. Cullinan et al. [8] and Roddy [9] surveyed families in Ireland and estimated the extra costs and losses of households with a chronic illness or disability. The results of this study showed that additional costs increased as the level of disability became more severe, as did the impact on poverty indicators. Minh et al. predicted the extra costs of living for households with a disability in Vietnam, noting that such additional costs come to around 8.8–9.5% of one’s annual household income (equating to USD 200–218) [10].

We referred to the work of Sado [11], who scrutinized the extra costs of depression—not only direct costs such as medical and pharmaceutical expenses, but also indirect ones such as morbidity costs (absenteeism and lost productivity due to depression) and mortality costs (expected lifetime wages of suicide victims who took their own lives due to depression).

Estimates of the extra costs of child abuse have been conducted in the US [12], Canada [13], Germany [14], Australia [15], and Japan. Our study supplements the work of Wada and Igarashi [1], as noted above.

## 2. Materials and Methods

### 2.1. Method of Selecting Facilities for Survey Distribution

The survey was designed by the second author of this paper and completed with advice from the first author. We delivered it to 260 facilities for CWD (welfare institutions) and received 91 responses. The initial plan was to send the survey to medical institutions as well. However, using budget as an indicator, the results of a pilot study of five medical institutions showed that it is difficult to compare the two types of facilities because they have vastly different goals (welfare institutions aim to improve children’s life skills, while medical institutions are focused on treating children). Thus, we did not send the survey to medical institutions. Accordingly, we only implemented the survey at welfare institutions. As support costs generally tend to be higher at medical institutions than at RCFs, the estimates are on the conservative side. Statistical data, such as budgets for welfare and medical institutions in Japan, are managed by local governments and are not compiled at the national level. The development of national welfare and medical statistics is still in its infancy in Japan. In addition, the central government does not fully disclose the small amount of statistical data that does exist, and an environment for secondary use by researchers is not yet in place.

We created the mailing list from addresses available in a national database. We sent out the survey in October 2020; the collection period ran until January 2021. We cleaned and analyzed the data using descriptive statistics and visualization.

### 2.2. Relationship between Recovery Rate and the Sample/Population

We received responses from 91 corporate facilities housing 2235 individuals (including children and overage children). According to data from MHLW’s DHWPD, there were 28,368 RCFs for CWD in 2020 (6944 welfare and 21,424 medical institutions). However, in this study, all surveys were answered by welfare RCFs for CWD. Thus, the sample comprised 2235 (32.1%) individuals living at 6944 welfare RCFs for CWD.

### 2.3. Ethical Review

The present study was approved by the Research Ethics Committee of Hanazono University. Those returning the survey form consented to this survey.

## 3. Results

### 3.1. Breakdown of Total Operating Expenses for the RCFs

As part of the survey, we gathered data on total operating expenses for each RCF as a whole, the budget allocated to children under 18 admitted to the facility based on the Child Welfare Act, and the budget allocated to overage children over 18 grounded in the Act on Providing Comprehensive Support for the Daily Lives and Life in Society of Persons with Disabilities. Extraordinary increases/decreases (which are receipts or spending other than direct support) are reported as expenses tied to the facility’s accounting department. Extraordinary increases/decreases include non-recurring grants, donations, and gains/losses on sales of fixed assets, as well as gains/losses on disposals of fixed assets.

**Figure 1:**
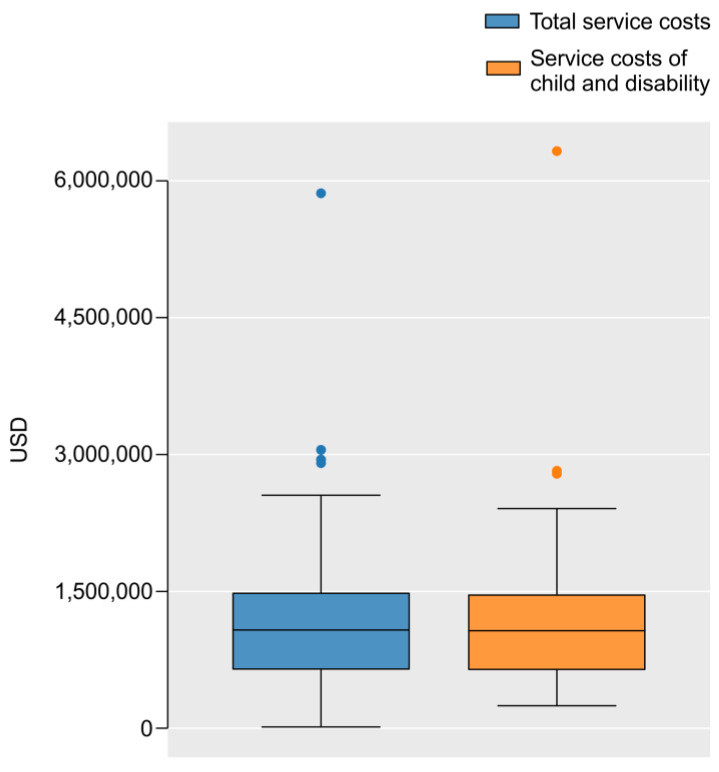
Total cost of services vs. direct support costs for children and people with disabilities.

The facility’s total operating expenses, which form the overall budget, averaged USD 1.17 million with SD ± 0.123 million; 96.7% of a facility’s total operating expenses are accounted for by government benefits through support services rooted in the Child Welfare Act and the Act on Providing Comprehensive Support for the Daily Lives and Life in Society of Persons with Disabilities (the Child Welfare Act budget + Act on Providing Comprehensive Support for the Daily Lives and Life in Society of Persons with Disabilities = the total cost; average: USD 1.14 million, SD ± 0.127 million) (Figure 1).

Under Japanese law, disabled children under age 18 have money allocated from the budget under the Child Welfare Act, while disabled persons over age 18 have money allocated from the Act on Providing Comprehensive Support for the Daily Lives and Life in Society of Persons with Disabilities. As we did not scrutinize the age of each individual resident, we determined there was no issue with each facility’s total operating expenses, which amounted to the sum of budgets for the Child Welfare Act and for the Act on Providing Comprehensive Support for the Daily Lives and Life in Society of Persons with Disabilities.

### 3.2. Cost Per Resident for an RCF for CWD and the Total Annual Amount

After we obtained the total operating expenses of each facility (Child Welfare Act budget + Act on Providing Comprehensive Support for the Daily Lives and Life in Society of Persons with Disabilities = the total cost), we divided the amount by the number of enrolled children (including overage children) and the annual direct support costs per resident. The average annual budget per child resident in welfare institutions for CWD was 31.7 (USD 1000) (95% confidence interval (CI)): 28.5 (USD 1000)–34.9 (USD 1000), the average annual budget per overage child was 25.5 (USD 1000) (95% CI: 12.4 (USD 1000)–38.7 (USD 1000)), and the average annual budget per resident in all facilities was 57.9 (USD 1000) (95% CI: 44.8 (USD 1000)–71.0 (USD 1000)) (Table 1).

All estimates are based on the costs of welfare institutions because data on medical institutions were not available. We may have underestimated the figures because medical institutions usually include spending from the medical care budget, in addition to the child welfare and disability welfare budgets.

Based on our estimates, the annual cost per child in RCFs for CWD is 31.7 (USD 1000) × 8727 child residents, and the annual cost per overage child is 25.5 (USD 1000) × 19,641 overage child residents, so the direct support costs of RCFs for CWD in Japan amounted to USD 777.4 million for the 2020 fiscal year.

### 3.3. Criteria for Child Abuse

In estimating the extra costs of abuse in RCFs for CWD, the criteria for identifying abuse—which factor into the reasons for admission to the facility—are important. However, it is difficult to pinpoint a single criterion (i.e., point estimation); it is more appropriate to adopt several different criteria and to consider the true values that lie within their range (partial identification) [16]. In this case, partial estimation is different from the confidence interval used to infer the population from a sample in statistical theory. There are multiple criteria for child abuse, but no dataset ensures the validity of the criteria currently used in Japan. Under these limited conditions, the attitude of conscientious science is to present multiple criteria and the possibility of a true value somewhere within an interval rather than point estimates of the true value.

The survey asked each facility about the number of children admitted whose disabilities were caused (or presumed to have been caused) by child abuse. We assumed this would be the criterion by which the child consultation center informed the facility that child abuse was considered to be the reason for admission. Accordingly, this criterion is referred to as the public criterion.

In addition, an MHLW research team’s 2019 survey of RCFs for CWD presented an estimate of “children for whom the child consultation center does not acknowledge abuse, but the facility judges to have been abused or strongly suspects to have been abused” [17] (p. 282). We adopted this estimate as the Shimoyamada criterion.

The survey also asked eligible facilities about the number of child residents with ACEs. In Japan, investigations of the relationship between ACEs and mental illness [18,19], PTSD [20,21], addictive behaviors [22], poverty [23], and delinquent/offending behavior [24] have been conducted. Although the ACE score is used to predict the frequency of deviant physical, mental, and social behaviors in adolescence and adulthood, we believe it can also be used for severe problems in the upbringing environment [25]; hence, we set two levels of applicability to ACE items in the survey. The ACE 1–4 criteria include experiences of (1) psychological abuse, (2) physical abuse, (3) sexual abuse, and (4) neglect, which are also close to the definition of child abuse in the Japanese Child Welfare Act.

In addition to the ACE 1–4 criteria, we defined the ACE 5–10 criteria as (5) the experience of feeling alienated, (6) the mother’s history of domestic violence, (7) divorce, (8) a family history of alcoholism, (9) a family history of mental illness, and (10) a family history of incarceration, all of which correspond to family dysfunction.

These four criteria are the public criterion, the Shimoyamada criterion, the ACE 1–4 criteria, and the ACE 5–10 criteria. The following table displays the percentages of children admitted that meet the criteria (Table 2). Therefore, in Japan, the true rate of children for whom abuse is a factor in their admission to an RCF for CWD is likely between 24.9% and 63.8%.

The reasons for adopting the four different criteria in this paper are as follows. In general, public agencies tend to estimate negative information such as child abuse low, while those who work directly with children in institutions are more willing to honestly report the severity of the effects of child abuse. We believe that the range of 23% to 67% for the four criteria is a difference in “position” between public agencies and support agencies. We base our estimates on the interval estimation idea that “the true value lies somewhere in between” rather than being biased one way or the other.

### 3.4. Calculating the Direct Costs of Child Abuse for RCFs for CWD

The rate of abuse among children admitted to RCFs for CWD was estimated to range from 24.9% to 63.8%. The rates of direct support costs for each of the four criteria (extra costs per person due to abuse) are shown in Table 3. The annual cost per person is USD 17.8 (USD 1000) based on the public criterion, USD 19.0 (USD 1000) based on the Shimoyamada criterion, USD 24.1 (USD 1000) based on the ACE 1–4 criteria, and USD 30.2 (USD 1000) based on the ACE 5–10 criteria (Table 3).

**Figure 2:**
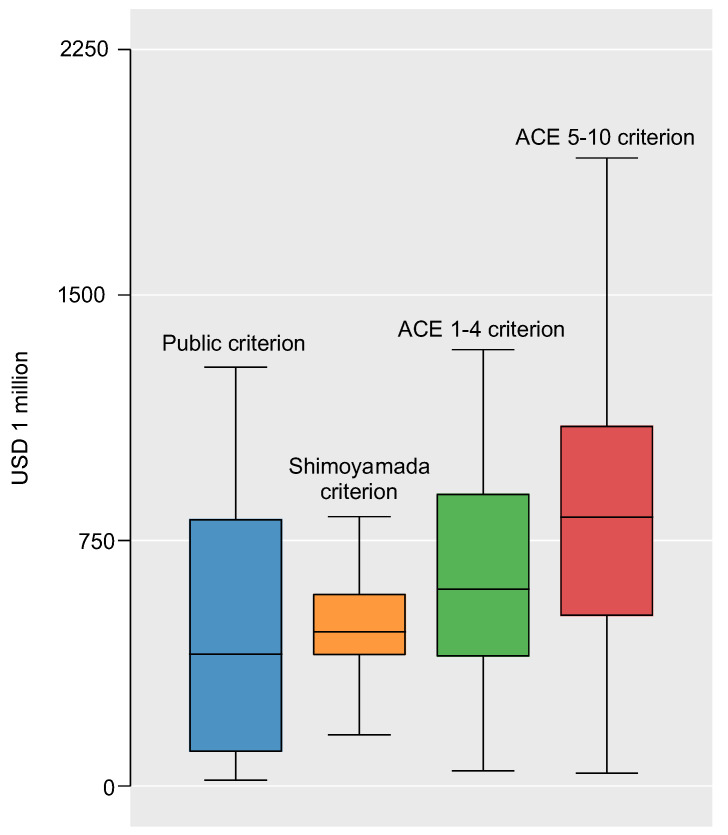
Box plot of estimated extra costs by each accreditation method (USD).

After multiplying the annual cost using the criteria by the number of residents of RCFs for CWD in 2020 (28,368), we found the extra costs due to child abuse to be USD 505.4 million according to the public criterion, USD 540 million according to the Shimoyamada criterion, USD 683 million according to the ACE 1–4 criteria, and USD 861.5 million according to the ACE 5–10 criteria. We therefore estimated the true value to be between USD 505.4 and USD 861.5 million. The average value of this partial identification, USD 647.7 million, was estimated to be the extra costs of child abuse for RCFs for CWD in this study (Table 4 and Figure 2).

In order to make a comparison with the findings of Wada and Igarashi, we estimated that the proportion of children alone (30%) would produce USD 194.3 million. This estimated amount is second only to the costs of abuse at USD 567 million for foster homes cited by Wada and Igarashi. Wada and Igarashi estimated direct costs to be USD 999 million at a time when estimates for RCFs for CWD were not included [1]. Adding the USD 647.7 million that we estimated, the extra costs due to child abuse in Japan amount to USD 1646.7 million.

## 4. Discussion

The results indicate that a minimum of 24.9% and maximum of 63.8% of the children admitted to RCFs for CWD were affected by child abuse in a broad sense. We would like to verify the validity of this estimate by adding additional samples and enhancing the official statistics in the future. Residents of RCFs for CWD might not just be congenitally delayed, developmentally disabled, or physically disabled; they may also have acquired impairments after the fact due to an inappropriate upbringing environment, or they might have PTSD (although judged to be an impairment) due to inappropriate care. This implies that RCFs for CWD substantively function as social care facilities, and may suffer from the same social problems as foster homes. In other words, whether due to a medical condition or abuse, under the circumstances of social care in Japan—where adoption and small-scale foster care are still developing—placement of CWD in institutions during childhood disconnects them from their families and communities, resulting in prolonged stays in facilities. This is supported by the phenomenon of children in RCFs for CWDs remaining there as overage children, even after they reach adulthood. According to MHLW [5], 39.2% transitioned to RCFs for persons with disabilities for adults, and 25.3% to assisted communal living (group homes) after leaving the facility, resulting in only a partial return to the home and community. As such, childhood institutionalization due to abuse may have a significant impact on adulthood.

This study estimated the extra costs related to the institutionalization of children that are presumably institutionalized because of child maltreatment, a topic on which there have been few research reports in recent years. Taking into account the differences across child welfare and social care systems, we would encourage future research on the extra costs of child maltreatment and associated institutionalization in areas other than Japan.

Although we only calculated the direct costs of residential care for CWD, we can infer from the results that the social costs of long-term admission—not only in residential care for CWD but also in foster homes and other facilities where children are separated from their families and communities—are far greater later in life than direct costs.

## 5. Conclusions

A minimum of 23% and a maximum of 67% of children in RCFs for CWD have been subjected to child abuse. Children admitted to residential care may have been post-disabled or have PTSD due to an inappropriate care environment. The direct costs of residential care for CWD 2020 amounted to USD 647.7 million, which is incurred annually. In the context of social care in Japan, where adoption and small-scale foster care are still in their infancy, entering institutions at an early age may cause CWD to be cut off from their families and communities, resulting in prolonged institutionalization. Subsequently, reintegration back into the family or community is not achieved, and residential care at an early age due to abuse has a negative impact on adulthood.

## Figures and Tables

**Table 1:** Estimated annual per capita budget for RCFs for CWD (USD 1000).

	Obs	Mean (USD 1000)	Std. Err.	(95% CI)	
Total service costs	74	57.9	6.6	44.8	71.0
Service costs for a child	76	31.7	1.6	28.5	34.9
Service costs for a disabled person	75	25.5	6.6	12.4	38.7

**Table 2:** Percentage of accreditation by each accreditation method.

	Percentage
Public criterion	24.9%
Shimoyamada criterion	39.9%
ACE 1–4 criteria	48.9%
ACE 5–10 criteria	63.8%

**Table 3:** Direct costs per person by each accreditation method (USD).

	Obs	Mean (USD 1000)	Std. Err.	(95% CI)	
Direct costs per person by the public criterion	56	17.8	2.7	12.4	23.3
Direct costs per person by the Shimoyamada criterion	74	19.0	1.1	16.9	21.2
Direct costs per person by the ACE 1–4 criteria	74	24.1	2.3	19.4	28.8
Direct costs per person by the ACE 5–10 criteria	73	30.2	2.7	24.7	35.8

Note. USD 1 = JPY 130.

**Table 4:** Estimated extra costs by each accreditation method (USD).

	Estimated Extra Costs (USD 1 Million)	Std. Err.	(95% CI)	
Total extra costs by the public criterion	505.4	76.4	350.8	660.8
Total extra costs by the Shimoyamada criterion	540.0	30.2	478.5	601.5
Total extra costs by the ACE 1–4 criteria	683.8	65.5	550.8	815.4
Total extra costs by the ACE 5–10 criteria	861.5	76.9	701.5	1015.4

Note. USD 1 = JPY 130.

## Data Availability

The data presented in this study are available on request from the corresponding author.

## References

[1] Wada I., Igarashi A. (2014). The social costs of child abuse in Japan. Child Youth Serv. Rev..

[2] Hughes K., Ford K., Bellis M.A., Glendinning F., Harrison E., Passmore J. (2021). Health and financial costs of adverse childhood experiences in 28 European countries: A systematic review and meta-analysis. Lancet Public Health.

[3] Isumi A., Fujiwara T., Kato H., Tsuji T., Takagi D., Kondo N., Kondo K. (2020). Assessment of additional medical costs among older adults in Japan with a history of childhood maltreatment. JAMA Netw. Open.

[4] Child Welfare Act (Act No. 164) 1947. https://www.japaneselawtranslation.go.jp/ja/laws/view/4035.

[5] Ministry of Health, Labour and Welfare, Department of Health and Welfare for Persons with Disabilities (2019). Residential Facilities for Children with Disabilities.

[6] Tsuru S. (2020). Practice and future direction of four functions of residential facilities for children with disabilities (Special issue: Secure children for the future) [in Japanese]. J. Intellec. Disabil. Welf..

[7] Suzuki H. (2020). Future direction of policies for children with disabilities: Findings from the study group on the state of residential facilities for disabled children. J. Intellec. Disabil. Welf..

[8] Cullinan J., Gannon B., Lyons S. (2011). Estimating the extra cost of living for people with disabilities. Health Econ..

[9] Roddy Á. (2022). Income and conversion handicaps: Estimating the impact of child chronic illness/disability on family income and the extra cost of child chronic illness/child disability in Ireland using a standard of living approach. Eur. J. Health Econ..

[10] Minh H.V., Giang K.B., Liem N.T., Palmer M., Thao N.P., Duong L.B. (2015). Estimating the extra cost of living with disability in Vietnam. Glob. Public Health.

[11] Sado M. (2014). Frontiers in psychiatry: What is the social cost of depression? Study on the cost of the illness of depression. Psychiatr. Neurol. Jpn..

[12] Miller T.R., Cohen M.A., Wiersema B. (1996). Victim Costs and Consequences: A New look.

[13] Bowlus A., McKenna K., Day T., Wright D. (2003). The Economic Costs and Consequences of Child Abuse in Canada.

[14] Meier-Gräwe U., Wagenknecht I. (2011). Expertise Kosten und Nutzen Früher.

[15] Taylor P., Moore P., Pezzullo L., Tucci J., Goddard C., De Bortoli L. (2008). The Cost of Child Abuse in Australia.

[16] Manski C.F. (2013). Public Policy in an Uncertain World: Analysis and Decisions.

[17] Shimoyamada Y. (2019). Survey on actual situation of abused children in facilities for children with disabilities: Children living in facilities, and children of short-term admission and temporary daycare. Jpn. J. Child Abuse Negl..

[18] Hiramatsu Y., Shimizu E. (2019). Effect of adverse childhood experiences on depression. Psychiatry.

[19] Tanaka E., Nishikawa M., Ohkubo K., Kameyama T. (2021). Adverse childhood experiences, lifetime trauma exposure, and PTSD in psychiatric clinics of Japan: A cross-sectional study. Psychiatr. Neurol. Jpn..

[20] Suzuki F., Makino T. (2020). Complex PTSD: Past and future. Psychiatry.

[21] Oe M. (2021). History and significance of diagnosis in complex PTSD: Including the issue of adverse childhood experiences. Psychotherapy.

[22] Itabashi T., Kobayashi S., Kurosawa F., Horiuchi E., Nakamura K., Hori S., Ohsone S., Inoue K., Kusuyama S. (2017). Classification of adverse childhood experiences of patients with substance use disorders. Jpn. J. Alcohol. Stud. Drug. Depend..

[23] Naka M. (2017). ‘ACEs’ and poverty: Eyes, ears, and mind to save children from adverse childhood experiences. Trends Sci..

[24] Hiroi I. (2020). Desirable perspectives on research on help-seeking of offenders: From the literature study of adverse childhood experiences and attachment theory. J. Clin. Educ..

[25] Wada I. (2018). Development of scientific assessment indicators for child abuse: Development of indicators to be used in decision-making for temporary protection, release, and measures for children. Proceedings of the Meiji Yasuda Mental Health Foundation Research Grant.

